# Deformation and Strength Parameters of a Composite Structure with a Thin Multilayer Ribbon-like Inclusion

**DOI:** 10.3390/ma15041435

**Published:** 2022-02-15

**Authors:** Volodymyr Hutsaylyuk, Yosyf Piskozub, Liubov Piskozub, Heorhiy Sulym

**Affiliations:** 1 Institute of Robots and Machine Design, Military University of Technology, Gen. S. Kaliskiego str. 2, 00-908 Warsaw, Poland; 2 Department of Applied Mathematics and Physics, Ukrainian Academy of Printing, Pidgolosko 19, 79020 L’viv, Ukraine; piskozub@pancha.lviv.ua (Y.P.); piskozub@uad.lviv.ua (L.P.); 3 Department of Mechanics and Applied Computer Science, Faculty of Mechanical Engineering, Bialystok University of Technology, Wiejska 45c, 15-351 Bialystok, Poland; h.sulym@pb.edu.pl

**Keywords:** functionally gradient material, composite, thin inhomogeneity, fracture mechanics, nonperfect contact, stress intensity factor

## Abstract

Within the framework of the concept of deformable solid mechanics, an analytical-numerical method to the problem of determining the mechanical fields in the composite structures with interphase ribbon-like deformable multilayered inhomogeneities under combined force and dislocation loading has been proposed. Based on the general relations of linear elasticity theory, a mathematical model of thin multilayered inclusion of finite width is constructed. The possibility of nonperfect contact along a part of the interface between the inclusion and the matrix, and between the layers of inclusion where surface energy or sliding with dry friction occurs, is envisaged. Based on the application of the theory of functions of a complex variable and the jump function method, the stress-strain field in the vicinity of the inclusion during its interaction with the concentrated forces and screw dislocations was calculated. The values of generalized stress intensity factors for the asymptotics of stress-strain fields in the vicinity of the ends of thin inhomogeneities are calculated, using which the stress concentration and local strength of the structure can be calculated. Several effects have been identified which can be used in designing the structure of layers and operation modes of such composites. The proposed method has shown its effectiveness for solving a whole class of problems of deformation and fracture of bodies with thin deformable inclusions of finite length and can be used for mathematical modeling of the mechanical effects of thin FGM heterogeneities in composites.

## 1. Introduction

Microscopic, layered structures in fields such as microelectronics, biotechnology, energy, weaponry, etc. are gaining special attention in modern engineering and technology. Among the most important scientific projects, experts identify a significant increase in computer performance, restoration of human organs using reproduced tissues (obtained from 3D printers) and obtaining new structured materials created directly from given molecules and atoms. Quite often these inclusions are used as elements to reinforce structural parts of machines and structures or as fillers of composite materials. Thin lamellar inhomogeneities are also a characteristic phenomenon at the interphase boundaries of crystalline grains arising during crystallization [1,2,3,4,5,6,7]. In this regard, there is a need to provide mathematical modeling of nanostructure mechanics, which is still a pressing problem of materials science theory. At this stage of the development of mechanics, it is already possible to concentrate on the construction of the complex universal equations suitable for investigations of multiscale, including layered, structures and the development of methods for their solution.

In such structures, each layer or their combination has its functional purpose, in particular, anti-corrosion, anti-abrasion, heat protection, strengthening to inhibit and block crack growth, reduce porosity, provide a high degree of adhesion of the components [8,9,10,11,12,13,14]. Thanks to multilayers, it is also possible to increase the service life of structures, and their use can significantly reduce material intensity and cost and increase the endurance of products. At the same time in a structure with thin layers, there is a concentration of stresses near places of change of physical and mechanical characteristics of materials. And it is the higher, the greater the difference in their properties.

Inhomogeneous structures with optimally varying physical and mechanical properties along with the thickness, known as functionally graded materials (FGMs) [15,16,17,18,19,20], allow one to reduce such stress concentrations in the vicinity of the contact between the matrix and the interlayer by avoiding abrupt transitions in the properties of the components. A detailed review of the manufacturing techniques can be found in [21,22,23,24,25,26]. FGMs are often used in the coatings of structural elements to protect them from the harmful effects of temperature [8,9,10,11,27,28,29,30,31,32,33,34,35], etc. One of the frequently used variants of FGM arrangement is the combination of ceramics with metal [36,37], but this often leads to the violation of the contact between them. Due to the brittle nature of ceramics, there is a need for additional research into the applicability limits of such FGM structures [38,39,40]. The complexity of the geometry of structural elements and consideration of imperfections in the contact of their components stimulate the process of improving mathematical models of FGMs to ensure their qualitative design both in terms of mechanical strength [12,13,14,41,42,43,44,45,46,47,48,49,50,51,52,53,54] and in terms of consideration of thermal, magnetic, piezoelectric loading factors [47,48]. The use of the FGMs seems to be one of the most effective materials in the realization of sustainable development in industries.

An important aspect of strength research, including tensile strength, for such structures, is to improve their strength criteria, to determine such key parameters as stress intensity factors (SIF) in the points of singularity. Moreover, since we consider thin inhomogeneities not only in the form of classical cracks but also thin cavities filled with an arbitrary elastic or nonlinearly elastic material, it makes sense to claim that the theory of thin inclusions is an essential generalization of the crack theory and the so-called generalized SIFs, which characterize the distribution of stress and displacement fields, are analogous parameters of fracture mechanics for the theory of thin inhomogeneities [55,56,57].

This work aims to develop an analytical and numerical method for studying the stress-strain state and strength of composites with thin deformable multilayer ribbon-like elements that are also suitable for mathematical modeling of thin inclusions with an almost arbitrary continuous thickness variation of mechanical characteristics.

## 2. Formulation of the Problem

**Figure 1:**
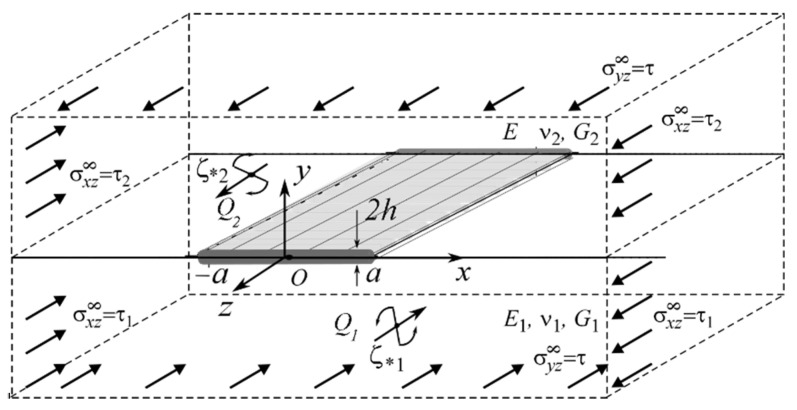
Geometry and load pattern of the problem.

**Figure 2:**
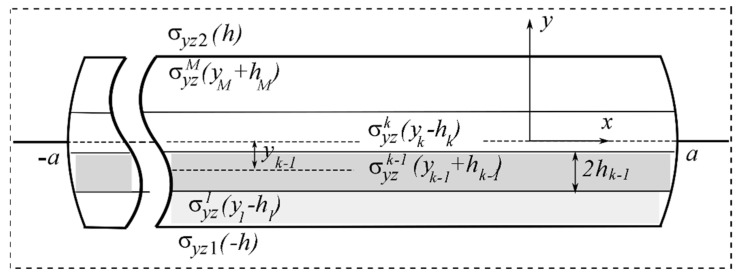
Multilayered inclusion.

We consider a structure which, following the concept of deformable solid mechanics, we will further consider as a combination of two half-spaces with elastic constants $E_{k},\nu_{k},\;G_{k}\;\left(k=1,2\right)$, at the interface of which (plane xOz) there is a tunnel section $L^{\prime }=\left[-a;\;a\right]$ in the direction of the shear axis Oz (Figure 1), in which a certain object of general thickness 2h(h≪a) is inserted–a package of M different thin plane-parallel layers $\left\{x\in L^{\prime };y\in \left[y_{K}-h_{K};y_{K}+h_{K}\right],K=\bar{1,M}\right\}$ of thickness $2h_{K}\left(2h=2\sum_{K=1}^{M}h_{K}\right),$ $y_{1}-h_{1}=-h,y_{M}+h_{M}=h$ with orthotropic mechanical properties $G_{y}^{inK},G_{x}^{inK}$ in the direction of two axes (Figure 2).

The structure is loaded quasi-statically by shear factors (uniform shear at infinity $\tau ,\tau_{k}$, concentrated forces $Q_{k}$, and screw dislocations $b_{k}$ at points $\varsigma_{k*}$), which cause longitudinal shear in the body. To ensure the straightness of the material interface at infinity, the stresses must satisfy the conditions $\sigma_{xz2}^{\infty }G_{1}=\sigma_{xz1}^{\infty }G_{2},$ $\frac{v_{2}\sigma_{yy}^{\infty }-(1-v_{2})\sigma_{xx2}^{\infty }}{G_{2}}=\frac{v_{1}\sigma_{yy}^{\infty }-(1-v_{1})\sigma_{xx1}^{\infty }}{G_{1}}$.

Let us restrict ourselves to the problem of longitudinal displacement in the direction of the *z*-axis (antiplane deformation). Then, considering that the stress-strain state (SSS) of the structure in each section perpendicular to the *z*-axis is identical, we will further consider only the plane xOy, which consists of two planar sections of half-spaces $S_{k}\left(k=1,2\right)$ with a separation boundary between them in the form of abscissa axes Ox.

## 3. Materials and Methods

The construction of a mathematical model of such a layered thin inclusion-layer (internal problem) should eventually reveal the relation between the stress-strain parameters inside the inclusion and on its external surface as the influence functions $\sigma_{yz}^{in}\left(x,\pm h\right),w^{in}\left(x,\pm h\right)$, which will be used in the further solution of the problem [51,52,53].

Let us introduce into consideration the jumps of the stress tensor components and the displacement vector for the matrix components and individual layers on $L_{}^{\prime }$:

(1) $$\begin{array}{l} {\left[\sigma_{yz}\right]}_{0,h}≅\sigma_{yz1}\left(x,-h\right)-\sigma_{yz}\left(x,h\right)=f_{3}\left(x\right),\\ {\left[\frac{\partial w}{\partial x}\right]}_{0,h}≅\frac{\partial w}{\partial x}\left(x,-h\right)-\frac{\partial w}{\partial x}\left(x,h\right)={\left[\frac{\sigma_{xz}}{G}\right]}_{0,h}=f_{6}\left(x\right),\;x\in L_{}^{\prime } \end{array}$$

(2) $$\begin{array}{l} {\left[\sigma_{yz}^{inK}\right]}_{y_{K},h_{K}}≅\sigma_{yz}^{inK}\left(x,y_{K}-h_{K}\right)-\sigma_{yz}^{inK}\left(x,y_{K}+h_{K}\right)=f_{3K}\left(x\right),\;\left(K=\bar{1,M}\right)\\ {\left[\frac{\partial w^{inK}}{\partial x}\right]}_{y_{K},h_{K}}≅\frac{\partial w^{inK}}{\partial x}\left(x,y_{K}-h_{K}\right)-\frac{\partial w^{inK}}{\partial x}\left(x,y_{K}+h_{K}\right)=f_{6K}\left(x\right),\;x\in L_{}^{\prime }; \end{array}$$

$f_{3}\left(x\right)=f_{6}\left(x\right)=0$, $f_{3K}\left(x\right)=f_{6K}\left(x\right)=0$, if $x\notin L^{\prime }$.

Hereinafter marked:

$${\left[•\right]}_{y,h}=•\left(x,y-h\right)-•\left(x,y+h\right),$$

$${\langle •\rangle }_{y,h}=•\left(x,y-h\right)+•\left(x,y+h\right).$$

Let us analyze the methodology for constructing a mathematical model for the case of a multilayer package of thin inclusion layers. The basic relation for an arbitrary orthotropic elastic material with shear moduli $G_{x}^{inK},G_{y}^{inK}$ of each of the layers given by the parameters $y_{K},h_{K}\left(K=\bar{1,M}\right)$ are the equilibrium conditions:

(3) $$\frac{\partial \sigma_{xz}^{inK}}{\partial x}+\frac{\partial \sigma_{yz}^{inK}}{\partial y}+\rho^{K}F_{}^{inK}=0,$$

where $\rho^{K}$ denotes a density of the material, and $F_{}^{inK}$—distribution of the mass forces, and constitutive strain-stress dependence (orthotropic linear elasticity):

(4) $$\sigma_{xz}^{inK}=G_{x}^{inK}\frac{\partial w^{inK}}{\partial x},\sigma_{yz}^{inK}=G_{y}^{inK}\frac{\partial w^{inK}}{\partial y}.$$

By integrating Equation (3) over the x limits [−a,x] and averaging, respectively, over the thicknesses of each of the heterogeneity layers $y\in \left[y_{K}-h_{K},y_{K}+h_{K}\right]$, we obtain:

(5) $$\frac{1}{2h_{K}}\int_{y_{K}-h_{K}}^{y_{K}+h_{K}}\sigma_{xz}^{inK}\left(\xi ,y\right)dy≃\frac{1}{2}\langle \sigma_{xz}^{inK}\rangle {}_{y_{K},h_{K}}=\frac{G_{x}^{inK}}{2}{\langle \frac{\partial w^{inK}}{\partial x}\rangle }_{y_{K},h_{K}},$$

and, accordingly, the first group of M equations of mathematical models of layers:

(6) $$\begin{array}{l} \frac{G_{x}^{inK}}{2}{\langle \frac{\partial w^{inK}}{\partial x}\rangle }_{y_{K},h_{K}}-\sigma_{xz}^{inK}\left(-a\right)-\frac{1}{2h_{K}}\int_{-a}^{x}{\left[\sigma_{yz}^{inK}\right]}_{y_{K},h_{K}}\left(\xi \right)d\xi +\\ +F_{aver}^{inK}\left(x,-h_{K},h_{K}\right)=0, \end{array}$$

where $F_{aver}^{inK}\left(x,-h_{K},h_{K}\right)=\frac{\rho^{K}}{2h_{K}}\int_{y_{K}-h_{K}}^{y_{K}+h_{K}}\int_{-a}^{x}F_{}^{inK}\left(\xi ,y\right)d\xi dy$, $\left(K=\bar{1,M}\right)$.

Considering the thin-wall ratio of the inclusion layers:

(7) $$\begin{array}{l} \frac{\partial w^{inK}}{\partial y}\left(x,y_{K}+h_{K}\right)+\frac{\partial w^{inK}}{\partial y}\left(x,y_{K}-h_{K}\right)≃\\ ≃\frac{w^{inK}\left(x,y_{K}+h_{K}\right)-w^{inK}\left(x,y_{K}-h_{K}\right)}{h_{K}}=-\frac{{\left[w^{inK}\right]}_{y_{K},h_{K}}}{h_{K}}, \end{array}$$

$$w^{inK}\left(x,y_{K}\mp h_{K}\right)=w^{inK}\left(x,y_{K}\pm h_{K}\right)\mp 2h_{K}\frac{\partial w^{inK}}{\partial y}\left(x,y_{K}\pm h_{K}\right)\left(K=\bar{1,M}\right),$$

and constitutive relations (4), we obtain the following form of the second group of M equations of the inclusion model:

(8) $$-\frac{{\left[w^{inK}\right]}_{y_{K},h_{K}}}{h_{K}}=\frac{\langle \sigma_{yz}^{inK}\rangle {}_{y_{K},h_{K}}}{G_{y}^{inK}}\left(K=\bar{1,M}\right),$$

which together with relations (6) fully describe the thin M -layered inclusion model written in the values of the stress-strain behavior of the inclusion package materials.

Instead of the displacement jump, in many cases, it is convenient to use the formula for the jump of the strain components:

$${\left[w^{inK}\right]}_{y_{K},h_{K}}\left(x\right)={\left[w^{inK}\right]}_{y_{K},h_{K}}\left(-a\right)+\int_{-a}^{x}{\left[\frac{\partial w^{inK}}{\partial x}\right]}_{y_{K},h_{K}}\left(\xi \right)d\xi .$$

At each inclusion layer, the balance conditions must be satisfied:

(9) $$\int_{-a}^{a}f_{3K}\left(\xi \right)d\xi =-N_{xzK}\left(-a\right)+N_{xzK}\left(a\right)+2hF_{averK}^{in}\left(a,h\right),$$

(10) $$\int_{-a}^{a}f_{6K}\left(\xi \right)d\xi ={\left[w^{inK}\right]}_{y_{K},h_{K}}\left(a\right)-{\left[w^{inK}\right]}_{y_{K},h_{K}}\left(-a\right),$$

where $N_{xzK}^{}(\pm a)=2h_{K}^{}\sigma_{\mathrm{xz}K}^{in}(\pm a)$.

The partial cases of the model (6), (8) of the form $\mu_{y}^{inK}=\mu_{y}^{in},\mu_{x}^{inK}=\mu_{x}^{in}\left(K=\bar{1,M}\right)$ (all layers are the same) or $h_{K}\to 0$ $\mu_{y}^{inK}=\mu_{y}^{in}\to 0,\mu_{x}^{inK}=\mu_{x}^{in}\to 0\left(K=\bar{1,M}\right)$ (no inclusion or crack) or $\mu_{y}^{inK}=\mu_{y}^{in}\to 0\left(G_{y}^{inK}=G_{y}^{in}\to \infty \right),$ $\mu_{x}^{inK}=\mu_{x}^{in}\to \infty \left(G_{x}^{inK}=G_{x}^{in}\to \infty \right)$ (perfectly rigid homogeneous inclusion) are satisfied and coincide with those known in the literature.

The solution for the matrix as an isotropic bimaterial (external problem) is obtained by the method of the problem of conjugation of analytic functions [51,52,53]:

(11) $$\begin{array}{l} \sigma_{sz}\left(x,y\right)=\sigma_{sz}^{0}\left(x,y\right)+{\hat{\sigma }}_{sz}\left(x,y\right),s=\left\{x,y\right\},\\ w\left(x,y\right)=w^{0}\left(x,y\right)+\hat{w}\left(x,y\right), \end{array}$$

(12) $$\begin{array}{l} \sigma_{yzk}\left(z\right)+i\sigma_{xzk}\left(z\right)=\sigma_{yzk}^{0}\left(z\right)+i\sigma_{xzk}^{0}\left(z\right)+ip_{k}g_{3}\left(z\right)-Cg_{6}\left(z\right)\\ \;\left(z\in S_{k};r=3,6;k=1,2\right),\\ \sigma_{yzk}^{\pm }\left(x\right)=\mp p_{k}f_{3}\left(x\right)-Cg_{6}\left(x\right)+\sigma_{yz}^{0\pm }\left(x\right),\\ \sigma_{xzk}^{\pm }\left(x\right)=\mp Cf_{6}\left(x\right)+p_{k}g_{3}\left(x\right)+\sigma_{xz}^{0\pm }\left(x\right),\\ {\frac{\partial w}{\partial y}}^{\pm }\left(x\right)=\mp pf_{3}\left(x\right)-p_{3-k}g_{6}\left(x\right)+\frac{\sigma_{yz}^{0\pm }\left(x\right)}{G_{k}},\\ {\frac{\partial w}{\partial x}}^{\pm }\left(x\right)=\mp p_{3-k}f_{3}\left(x\right)+pg_{6}\left(x\right)+\frac{\sigma_{xz}^{0\pm }\left(x\right)}{G_{k}}, \end{array}$$

where:

$$g_{r}\left(z\right)\equiv \frac{1}{\pi }\int_{L_{}^{\prime }}\frac{f_{r}\left(x\right)dx}{x-z}\;,\;s_{r}\left(x\right)\equiv \int_{-a}^{x}f_{r}\left(x\right)dx,C=G_{3-k}p_{k},\;p_{k}=pG_{k},p=\frac{1}{G_{1}+G_{2}}.$$

Here the upper indexes “+” and “−” correspond to the limit values of the functions at the upper and lower margins of the line L; the values marked with the index “0” on the top characterize the corresponding values in a solid body without modeled heterogeneities under the corresponding external load, and the values marked with the symbol “^” on the top, are the perturbations of the basic stress-strain field by the presence of an inclusion [53].

The following entries [47,49] shall continue to apply:

$$\begin{array}{l} \sigma_{yz}^{0}\left(z\right)+i\sigma_{xz}^{0}\left(z\right)=\tau +i\{\tau_{k}+D_{k}\left(z\right)+\left(p_{k}-p_{j}\right){\bar{D}}_{k}\left(z\right)+2p_{k}D_{j}\left(z\right)\},\\ D_{k}\left(z\right)=-\frac{Q_{k}+iG_{k}b_{k}}{2\pi \left(z-\varsigma_{k*}\right)}\;\left(z\in S_{k},\;k=1,2;\;j=3-k\right). \end{array}$$

To connect the external and internal problems, one needs to use contact conditions between the components of the package. There are several variants of contact conditions between the layers and between the package and the matrix:

(1) Ideal (perfect) contact between all constituents of the package:

(13) $$\{\begin{array}{l} w^{in\left(K-1\right)}\left(x,y_{K-1}+h_{K-1}\right)=w^{inK}\left(x,y_{K}-h_{K}\right)\left(K=\bar{2,M}\right),\\ \sigma_{yz}^{in\left(K-1\right)}\left(x,y_{K-1}+h_{K-1}\right)=\sigma_{yz}^{inK}\left(x,y_{K}-h_{K}\right)\left(x\in L^{\prime }\right). \end{array}$$

(2) Nonperfect contact with additional tension between layers:

(14) $$\{\begin{array}{l} w^{in\left(K-1\right)}\left(x,y_{K-1}+h_{K-1}\right)=w^{inK}\left(x,y_{K}-h_{K}\right)\left(K=\bar{2,M}\right),\\ \sigma_{yz}^{inK}\left(x,y_{K}-h_{K}\right)=\sigma_{yz}^{in\left(K-1\right)}\left(x,y_{K-1}+h_{K-1}\right)-T_{K}. \end{array}$$

$T_{K}$ are the surface stresses. When $T_{K}=0$ we have the same ideal contact (13).

(3) Contact with friction between the (K)-th and (K−1)-th layers at the boundary $\left\{x,y_{K}\pm h_{K}\right\}$ in some area $x\in L_{f}\subset L^{\prime }$

(15) $$\sigma_{yz}^{in\left(K-1\right)}\left(x,y_{K-1}+h_{K-1}\right)=\sigma_{yz}^{inK}\left(x,y_{K}-h_{K}\right)=-sgn{\left[w^{in}\right]}_{y_{K},h_{K}}\tau_{yzK}^{max}.$$

$\tau_{yzK}^{max}$ are the limit value of tangential stresses, at which slippage begins. When $\tau_{yzK}^{max}$ there is a smooth contact between these layers.

(4) Ideal contact between the boundary components of the package and the matrix:

(16) $$\{\begin{array}{l} w^{in1}\left(x,y_{1}-h_{1}\right)=w_{}\left(x,y_{1}-h_{1}\right)\\ \sigma_{yz}^{in1}\left(x,y_{1}-h_{1}\right)=\sigma_{yz1}\left(x,y_{1}-h_{1}\right),\\ w^{inM}\left(x,y_{M}+h_{M}\right)=w_{}\left(x,y_{M}+h_{M}\right),\\ \sigma_{yz}^{inK}\left(x,y_{M}+h_{M}\right)=\sigma_{yz2}\left(x,y_{M}+h_{M}\right)\left(x\in L^{\prime }\right). \end{array}$$

(5) Nonperfect contact between the edge components of the package and the matrix:

(17) $$\{\begin{array}{l} w^{in1}\left(x,y_{1}-h_{1}\right)=w_{}\left(x,y_{1}-h_{1}\right)\\ \sigma_{yz}^{in1}\left(x,y_{1}-h_{1}\right)=\sigma_{yz1}\left(x,y_{1}-h_{1}\right)-T_{1},\\ w^{inM}\left(x,y_{M}+h_{M}\right)=w_{}\left(x,y_{M}+h_{M}\right),\\ \sigma_{yz}^{inM}\left(x,y_{M}+h_{M}\right)=\sigma_{yz2}\left(x,y_{M}+h_{M}\right)+T_{M+1}\left(x\in L^{\prime }\right). \end{array}$$

In the case $T_{1}=T_{M+1}=0$ we have an ideal contact (16).

(6) Contact with friction between the inclusion and the matrix within $\left\{x,y_{1}-h_{1}\right\}$, $\left\{x,y_{M}+h_{M}\right\}$ in some area $x\in L_{f}\subset L^{\prime }$

(18) $$\{\begin{array}{l} \sigma_{yz1}\left(x,y_{1}-h_{1}\right)=\sigma_{yz}^{in1}\left(x,y_{1}-h_{1}\right)=-sgn{\left[w^{in}\right]}_{y_{1},h_{1}}\tau_{yz1}^{max}\\ \sigma_{yz2}\left(x,y_{M}+h_{M}\right)=\sigma_{yz}^{inM}\left(x,y_{M}+h_{M}\right)=-sgn{\left[w\right]}_{y_{M},h_{M}}\tau_{yzM}^{max}\{\left(x\in L_{f}\right) \end{array}$$

In the case $\tau_{yzK}^{max}$ there is a smooth contact.

One of the conditions (13)–(15) and one of the conditions (16)–(18) must be fulfilled simultaneously.

Using (2) and, for example, (14), we can obtain the expressions for the stresses and strains in the layers through the stress and strain limits for the inclusion package for the presence of interlayer tension:

(19) $$\begin{array}{l} \sigma_{yz}^{inK}\left(x,y_{K}+h_{K}\right)=\sigma_{yz}^{in1}\left(x,-h\right)-\overset{K}{\underset{j=1}{\sum }}f_{3,j}-\overset{K}{\underset{j=2}{\sum }}T_{j}=\sigma_{yz}^{inM}\left(x,h\right)+\overset{M}{\underset{j=K+1}{\sum }}f_{3,j}+\overset{M}{\underset{j=K+1}{\sum }}T_{j},\\ \frac{\partial w^{inK}}{\partial x}\left(x,y_{K}+h_{K}\right)=\frac{\partial w^{in1}}{\partial x}\left(x,-h\right)-\overset{K}{\underset{j=1}{\sum }}f_{6,j}=\frac{\partial w^{inM}}{\partial x}\left(x,h\right)+\overset{M}{\underset{j=K+1}{\sum }}f_{6,j}\left(x\in L^{\prime }\right). \end{array}$$

Here, the total jumps of the boundary stresses and strains for the inclusion package have the value:

(20) $$\begin{array}{l} \sigma_{yz}^{in1}\left(x,-h\right)-\sigma_{yz}^{inM}\left(x,h\right)=\overset{M}{\underset{j=1}{\sum }}f_{3,j}+\overset{M}{\underset{j=2}{\sum }}T_{j},\\ \frac{\partial w^{in1}}{\partial x}\left(x,-h\right)-\frac{\partial w^{inM}}{\partial x}\left(x,h\right)=\overset{M}{\underset{j=1}{\sum }}f_{6,j}\left(x\in L^{\prime }\right). \end{array}$$

The resulting limit stresses and strains for the inclusion package (19), the boundary values of the stresses and strains of the matrix (12), and the boundary conditions (13)–(18) form a complete system of singular integral equations (SSIE) for the solution of the problem. Note that the dissimodularity of the inclusion layers in no way affects the peculiarity of the solution of the SSIE of the problem, which allows us to obtain a large variety of effects from manipulating the properties of the layers.

**Figure 3:**
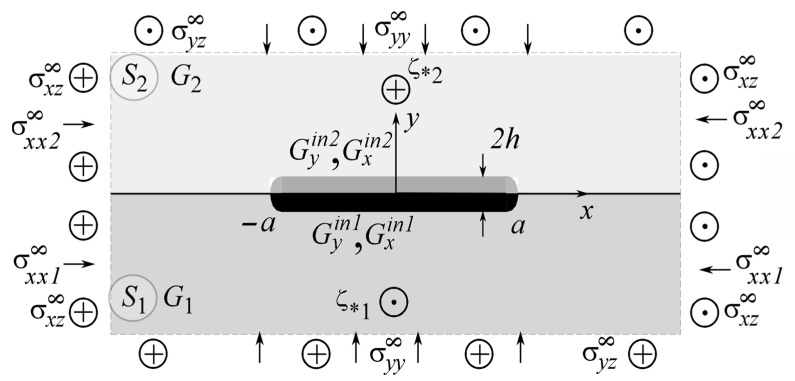
Geometry and load pattern of the problem for two-layer different-modularity thin inclusion.

To illustrate the method, let us investigate the longitudinal shear of a structure in the form of a body with a thin two-layer inclusion with layers of thickness $2h_{K}\left(K=1,2\right),\;2h=2h_{1}+2h_{2}$ and orthotropic mechanical properties $G_{y}^{inK},G_{x}^{inK}$, respectively, under the condition of nonperfect mechanical contact with surface tension on the contact surfaces of the structural components under different kinds of loading (Figure 3) when $\varsigma_{k*}=x_{k*}+iy_{k*}\left(k=1,2\right)$.

According to (6), (8), the mathematical model for the two-layer inclusion is as follows:

(21) $$\{\begin{array}{l} \mu_{x}^{in1}{\langle \frac{\partial w^{in1}}{\partial x}\rangle }_{y_{1},h_{1}}\left(x\right)-\int_{-a}^{x}{\left[\sigma_{xz}^{in1}\right]}_{y_{1},h_{1}}\left(\xi \right)d\xi =2h_{1}\left(\sigma_{xz}^{in1}\left(-a\right)-F_{aver}^{in1}\left(x\right)\right),\\ \mu_{x}^{in2}{\langle \frac{\partial w^{in2}}{\partial x}\rangle }_{y_{2},h_{2}}\left(x\right)-\int_{-a}^{x}{\left[\sigma_{xz}^{in2}\right]}_{y_{2},h_{2}}\left(\xi \right)d\xi =2h_{2}\left(\sigma_{xz}^{in2}\left(-a\right)-F_{aver}^{in2}\left(x\right)\right),\\ \mu_{y}^{in1}\langle \sigma_{yz}^{in1}\rangle {}_{y_{1},h_{1}}+\int_{-a}^{x}{\left[\frac{\partial w_{}^{in1}}{\partial x}\right]}_{y_{1},h_{1}}d\xi +{\left[w^{in1}\right]}_{y_{1},h_{1}}\left(-a\right)=0,\\ \mu_{y}^{in2}\langle \sigma_{yz}^{in2}\rangle {}_{y_{2},h_{2}}+\int_{-a}^{x}{\left[\frac{\partial w_{}^{in2}}{\partial x}\right]}_{y_{2},h_{2}}d\xi +{\left[w^{in2}\right]}_{y_{2},h_{2}}\left(-a\right)=0. \end{array}$$

The boundary conditions between the surfaces of the layers and the tunnel section on $L^{\prime }=\left[-a;\;a\right]$ take nonperfect with surface tension (14)–(17):

(22) $$\begin{array}{l} \sigma_{yz}^{in1}\left(x,y_{1}-h_{1}\right)=\sigma_{yz1}\left(x,-h\right)-T_{1},\\ \sigma_{yz}^{in2}\left(x,y_{2}-h_{2}\right)=\sigma_{yz1}^{in1}\left(x,y_{1}+h_{1}\right)-T_{2},\\ \sigma_{yz2}\left(x,h\right)=\sigma_{yz}^{in2}\left(x,y_{2}+h_{2}\right)-T_{3},\\ w\left(x,-h\right)=w_{}^{in1}\left(x,y_{1}-h_{1}\right),\\ w_{}^{in2}\left(x,y_{2}-h_{2}\right)=w_{}^{in1}\left(x,y_{1}+h_{1}\right),\\ w_{}^{in2}\left(x,y_{2}+h_{2}\right)=w\left(x,h\right). \end{array}$$

Given the boundary conditions (22), the relations between jumps (1) and (2) take the form:

(23) $$\begin{array}{l} f_{3}\left(x\right)=f_{3,1}\left(x\right)+f_{3,2}\left(x\right)+T_{1}+T_{2}+T_{3},\\ f_{6}\left(x\right)=f_{6,1}\left(x\right)+f_{6,2}\left(x\right). \end{array}$$

In addition, the integral representations of the external problem (11), (12), if the tension $T_{K}$ is not a function of the *Ox* coordinate, can be written as:

(24) $$\begin{array}{l} \sigma_{yz2}\left(x,h\right)=-p_{2}f_{3,1}\left(x\right)-p_{2}f_{3,2}\left(x\right)-Cg_{6,1}\left(x\right)-Cg_{6,2}\left(x\right)-\\ -p_{2}\left(T_{1}+T_{2}+T_{3}\right)+\sigma_{yz}^{0+}\left(x\right),\\ \sigma_{yz1}\left(x,-h\right)=p_{1}f_{3,1}\left(x\right)+p_{1}f_{3,2}\left(x\right)-Cg_{6,1}\left(x\right)-Cg_{6,2}\left(x\right)+\\ +p_{1}\left(T_{1}+T_{2}+T_{3}\right)+\sigma_{yz}^{0-}\left(x\right),\\ {\frac{\partial w}{\partial x}}^{}\left(x,h\right)=-p_{1}f_{6,1}\left(x\right)-p_{1}f_{6,2}\left(x\right)+pg_{6,1}\left(x\right)+pg_{6,2}\left(x\right)+\\ +\frac{p}{\pi }\left(T_{1}+T_{2}+T_{3}\right)ln\left|\frac{1-x}{1+x}\right|+\frac{\sigma_{xz}^{0+}\left(x\right)}{G_{2}},\\ {\frac{\partial w}{\partial x}}^{}\left(x,-h\right)=p_{2}f_{6,1}\left(x\right)+p_{2}f_{6,2}\left(x\right)+pg_{6,1}\left(x\right)+pg_{6,2}\left(x\right)+\\ +\frac{p}{\pi }\left(T_{1}+T_{2}+T_{3}\right)ln\left|\frac{1-x}{1+x}\right|+\frac{\sigma_{xz}^{0-}\left(x\right)}{G_{1}}, \end{array}$$

which agrees well with (23).

Substituting (22)–(24) into (21) considering the expressions:

(25) $$\begin{array}{l} \sigma_{yz1}^{in1}\left(x,y_{1}-h_{1}\right)=\sigma_{yz1}\left(x,-h\right)-T_{1},\\ \sigma_{yz2}^{in2}\left(x,y_{2}+h_{2}\right)=\sigma_{yz2}\left(x,h\right)+T_{3},\\ \sigma_{yz1}^{in1}\left(x,y_{1}+h_{1}\right)=\sigma_{yz}^{in2}\left(x,y_{2}-h_{2}\right)+T_{2}=\\ =\sigma_{yz2}^{in2}\left(x,y_{2}+h_{2}\right)+f_{3,2}\left(x\right)+T_{2}=\\ =\sigma_{yz2}\left(x,h\right)+f_{3,2}\left(x\right)+T_{2}+T_{3},\\ \sigma_{yz2}^{in2}\left(x,y_{2}-h_{2}\right)=\sigma_{yz1}^{in1}\left(x,y_{1}+h_{1}\right)-T_{2}=\\ =\sigma_{yz1}^{in1}\left(x,y_{1}-h_{1}\right)-f_{3,1}\left(x\right)-T_{2}=\\ =\sigma_{yz1}\left(x,-h\right)-f_{3,1}\left(x\right)-T_{2}-T_{1}, \end{array}$$

generates the following kind of two-layer multi-module thin inclusion model in terms of jumps:

(26) $$\{\begin{array}{l} \left(p_{2}-p_{1}\right)f_{6,1}\left(x\right)+2p_{2}f_{6,1}\left(x\right)+2pg_{3,1}\left(x\right)+2pg_{3,2}\left(x\right)-\frac{1}{\mu_{x}^{in1}}\int_{-a}^{x}f_{3,1}\left(\xi \right)d\xi =\\ =\frac{2h_{1}}{\mu_{x}^{in1}}\left(\sigma_{xz}^{in1}\left(-a\right)-F_{aver}^{in1}\left(x\right)\right)-{\langle \frac{\partial w^{0}}{\partial x}\rangle }_{h}\left(x\right)-\left\{T_{1}+T_{2}+T_{3}\right\}\frac{2p}{\pi }ln\left|\frac{1-x}{1+x}\right|,\\ -2p_{1}f_{6,1}\left(x\right)+\left(p_{2}-p_{1}\right)f_{6,2}\left(x\right)+2pg_{3,1}\left(x\right)+2pg_{3,2}\left(x\right)-\frac{1}{\mu_{x}^{in2}}\int_{-a}^{x}f_{3,2}\left(\xi \right)d\xi =\\ =\frac{2h_{2}}{\mu_{x}^{in2}}\left(\sigma_{xz}^{in2}\left(-a\right)-F_{aver}^{in2}\left(x\right)\right)-{\langle \frac{\partial w^{0}}{\partial x}\rangle }_{h}\left(x\right)-\left\{T_{1}+T_{2}+T_{3}\right\}\frac{2p}{\pi }ln\left|\frac{1-x}{1+x}\right|,\\ -\left(p_{2}-p_{1}\right)f_{3,1}\left(x\right)+2p_{1}f_{3,2}\left(x\right)-2Cg_{6,1}\left(x\right)-2Cg_{6,2}\left(x\right)+\frac{1}{\mu_{y}^{in1}}\int_{-a}^{x}f_{6,1}\left(\xi \right)d\xi =\\ \;\;\;=-\frac{1}{\mu_{y}^{in1}}{\left[w^{in1}\right]}_{y_{1},h_{1}}\left(-a\right)+T_{1}-T_{2}-T_{3}-\langle \sigma_{yzk}^{0}\rangle {}_{h}\left(x\right),\\ -2p_{2}f_{3,1}\left(x\right)-\left(p_{2}-p_{1}\right)f_{3,2}\left(x\right)-2Cg_{6,1}\left(x\right)-2Cg_{6,2}\left(x\right)+\frac{1}{\mu_{y}^{in2}}\int_{-a}^{x}f_{6,2}\left(\xi \right)d\xi =\\ \;\;\;=-\frac{1}{\mu_{y}^{in2}}{\left[w^{in2}\right]}_{y_{2},h_{2}}\left(-a\right)+T_{1}+T_{2}-T_{3}-\langle \sigma_{yzk}^{0}\rangle {}_{h}\left(x\right). \end{array}$$

The resulting SSIE is supplemented by additional balance conditions:

(27) $$\int_{-a}^{a}f_{3,1}\left(\xi \right)d\xi =N_{xz}^{in1}\left(a\right)-N_{xz}^{in1}\left(-a\right)+a\left(T_{1}+T_{2}\right),\;\int_{-a}^{a}f_{3,2}\left(\xi \right)d\xi =N_{xz}^{in2}\left(a\right)-N_{xz}^{in2}\left(-a\right)+a\left(T_{2}+T_{3}\right),$$

(28) $$\int_{-a}^{a}f_{6,1}\left(\xi \right)d\xi ={\left[w^{in1}\right]}_{y_{1},h_{1}}\left(a\right)-{\left[w^{in1}\right]}_{y_{1},h_{1}}\left(-a\right),\;\int_{-a}^{a}f_{6,2}\left(\xi \right)d\xi ={\left[w^{in2}\right]}_{y_{2},h_{2}}\left(a\right)-{\left[w^{in2}\right]}_{y_{2},h_{2}}\left(-a\right),$$

where $N_{xz}^{inK}\left(x\right)=2h_{K}\left(\sigma_{xz}^{inK}\left(-a\right)-F_{aver}^{inK}\left(x\right)\right)\left(K=1,2\right)$.

or:

(29) $$\begin{array}{l} \int_{-a}^{a}f_{3}\left(\xi \right)d\xi =N_{xz}^{in}\left(a\right)-N_{xz}^{in}\left(-a\right)+a\left(T_{1}+T_{3}\right),\\ \int_{-a}^{a}f_{3,1}\left(\xi \right)d\xi +\int_{-a}^{a}f_{3,2}\left(\xi \right)d\xi +2a\left(T_{1}+T_{2}+T_{3}\right)=\\ =N_{xz}^{in1}\left(a\right)-N_{xz}^{in1}\left(-a\right)+a\left(T_{1}+T_{2}\right)+N_{xz}^{in2}\left(a\right)-N_{xz}^{in2}\left(-a\right)+a\left(T_{2}+T_{3}\right), \end{array}$$

$$\int_{-a}^{a}f_{3,1}\left(\xi \right)d\xi +\int_{-a}^{a}f_{3,2}\left(\xi \right)d\xi =N_{xz}^{in1}\left(a\right)-N_{xz}^{in1}\left(-a\right)+N_{xz}^{in2}\left(a\right)-N_{xz}^{in2}\left(-a\right)-a\left(T_{1}+T_{3}\right).$$

To preserve the quasi-static equilibrium of the considered microstructure, one should also require the fulfillment of the condition of the balance of surface forces $T_{1}+T_{3}=2T_{2}$.

The resulting system of Equations (26)–(29) is reduced to a system of linear algebraic equations concerning the unknown coefficients of the decomposition of the desired influence functions [51,52,53] into a series by Jacobi-Chebyshev polynomials, described in [55].

## 4. Numerical Results and Discussion

In fracture mechanics, it is common to use the stress intensity factor (SIF) $K_{3}$ to describe the asymptotics of the SSS in the vicinity of the crack tip [8,38]. For the case of a thin elastic inclusion, this is not sufficient [56]. The introduction of a system of polar coordinates (r,θ) with the origin near the right or the left tip of the inclusion $z_{1}=\pm rexp(i\theta )\pm a$ (Figure 3), makes it possible to obtain two-term asymptotic expressions for the distribution of the stresses and displacements in the vicinity of the tips $\left(\left|z_{1}\right|\ll 2a\right)$ [55] using the generalized stress intensity factors (GSIF) introduced by the expression:

$$K_{31}+iK_{32}=\underset{r\to \infty \left(\theta =0,\pi \right)}{lim}\sqrt{2\pi r}\left(\sigma_{yz}+i\sigma_{xz}\right).$$

Consider also the following dimensionless values, marked with a “~” at the top, which significantly reduce the number of calculations without loss in generality:

(30) $$\begin{array}{l} \tilde{x}=\frac{x}{a,}{\tilde{h}}_{K}=\frac{h_{K}}{a,}\tilde{y}=\frac{y}{a},{\tilde{\tau }}_{k}=\frac{\tau_{k}}{G_{gav}},\tilde{\tau }=\frac{\tau }{G_{gav}},\\ {\tilde{G}}_{x}^{inK}\left(\tilde{x}\right)=\frac{G_{x}^{inK}\left(x\right)}{G_{gav},}{\tilde{G}}_{y}^{inK}\left(\tilde{x}\right)=\frac{G_{y}^{inK}\left(x\right)}{G_{gav}},\\ {\tilde{G}}_{k}=\frac{G_{k}}{G_{gav}\left(k=1,2\right),}{\tilde{p}}_{k}=p_{k},\tilde{C}=\frac{C}{G_{gav},}\\ {\tilde{\sigma }}_{xz}\left(\tilde{x}\right)=\frac{{\tilde{\sigma }}_{xz}\left(x\right)}{G_{gav},}{\tilde{\sigma }}_{yz}\left(\tilde{x}\right)=\frac{{\tilde{\sigma }}_{yz}\left(x\right)}{G_{gav}},G_{gav}=\sqrt{G_{1}G_{2}}. \end{array}$$

(31) $${\tilde{K}}_{31}=\frac{K_{31}^{+}}{2\tilde{C}G_{gav}\sqrt{\pi a}},\;{\tilde{K}}_{32}=\frac{K_{32}^{+}}{2{\tilde{p}}_{2}G_{gav}\sqrt{\pi a}},$$

where $K_{31}^{+}$, $K_{32}^{+}$—GSIF’s near the tip of inclusion *x* = +*a*.

**Figure 4:**
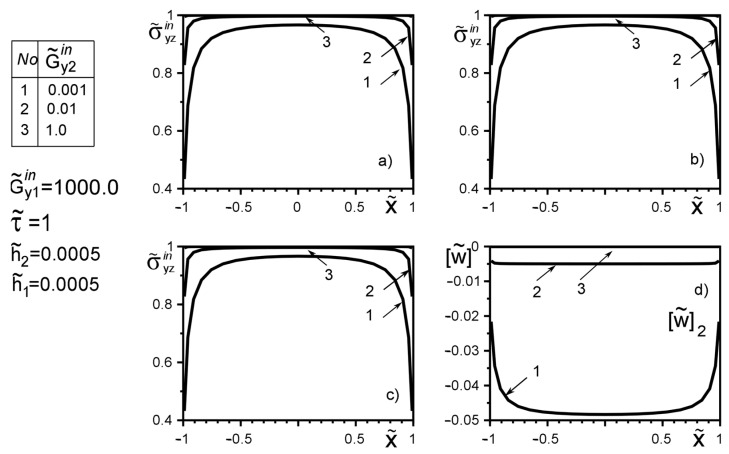
Stress distribution along with the upper interface (layer 2 of the inclusion—matrix half-space $S_{2}$) (**a**); the boundary between layers (layer 1–layer 2) (**b**); lower interface (layer 1—matrix half-space $S_{1}$ ) (**c**), and the displacement jump on the inclusion (**d**) for a layer 1 stiffer than the matrix as a function of the change in stiffness of layer 2 under the load uniformly distributed at infinity.

**Figure 5:**
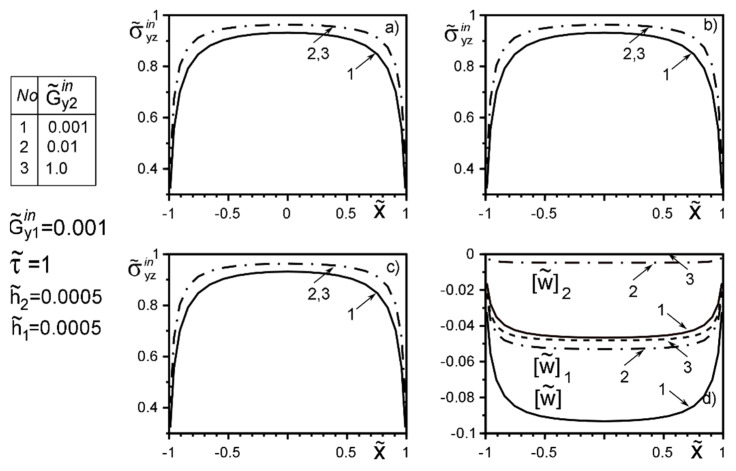
Stress distribution along with the upper interface (layer 2 of the inclusion—matrix half-space $S_{2}$) (**a**); the boundary between layers (layer 1–layer 2) (**b**); lower interface (layer 1—matrix half-space $S_{1}$ ) (**c**), and the displacement jump on the inclusion (**d**) for a layer 1 softer than the matrix as a function of the change in stiffness of layer 2 under the load uniformly distributed at infinity (1—result for the case of the same layer materials, verified by comparison with [50,53]).

**Figure 6:**
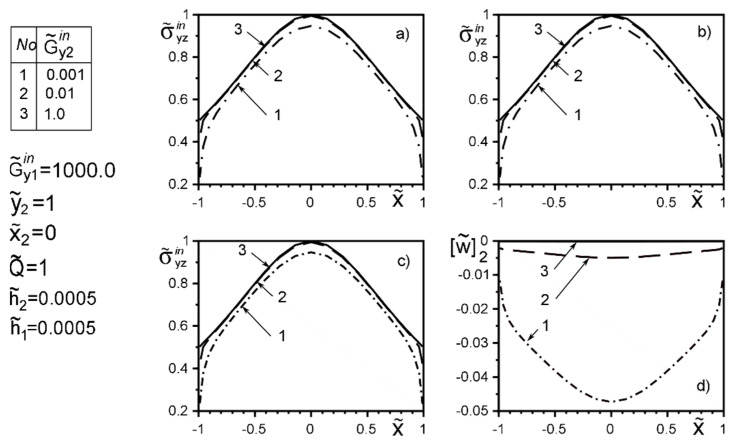
Stress distribution along with the upper interface (layer 2 of the inclusion–matrix half-space $S_{2}$) (**a**); the boundary between layers (layer 1–layer 2) (**b**); lower interface (layer 1—matrix half-space $S_{1}$ ) (**c**), and the displacement jump on the inclusion (**d**) for a layer 1 stiffer than the matrix as a function of the change in stiffness of layer 2 under the load by concentrated forces at points $\varsigma_{k*}=x_{k*}+iy_{k*}$; $x_{k*}=0,y_{2*}=-y_{1*}=d$.

**Figure 7:**
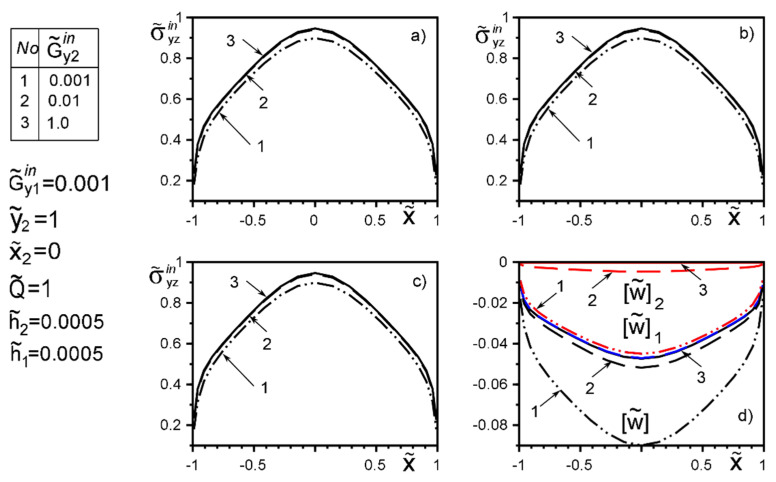
Stress distribution along with the upper interface (layer 2 of the inclusion–matrix half-space $S_{2}$) (**a**); the boundary between layers (layer 1–layer 2) (**b**); lower interface (layer 1—matrix half-space $S_{1}$ ) (**c**), and the displacement jump on the inclusion (**d**) for a layer 1 softer than the matrix as a function of the change in stiffness of layer 2 under the load by concentrated forces at points $\varsigma_{k*}=x_{k*}+iy_{k*}$; $x_{k*}=0,y_{2*}=-y_{1*}=d$ (1—result for the case of the same layer materials, verified by comparison with [50,53]).

**Figure 8:**
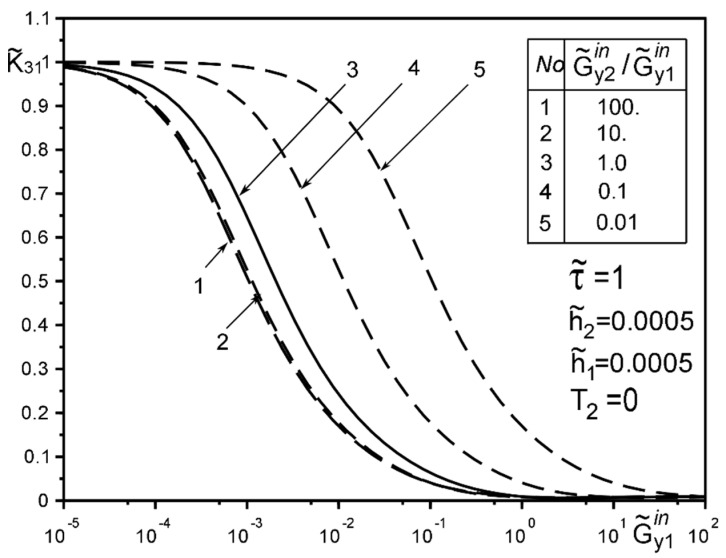
Influence of the level of dissimodularity on the GSIF ${\tilde{K}}_{31}$ under the load by uniformly distributed on infinity stress and absence of surface tension.

**Figure 9:**
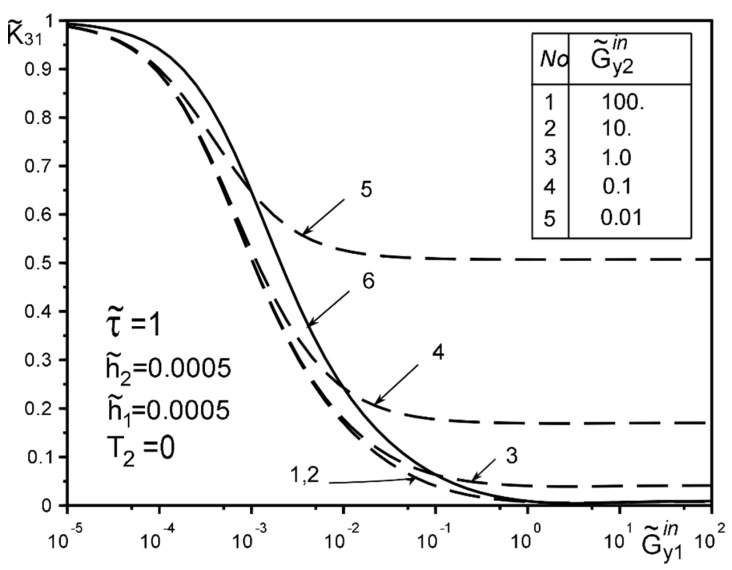
Influence of the level of dissimodularity on the GSIF ${\tilde{K}}_{31}$ under the load by uniformly distributed on infinity stress and absence of surface tension.

**Figure 10:**
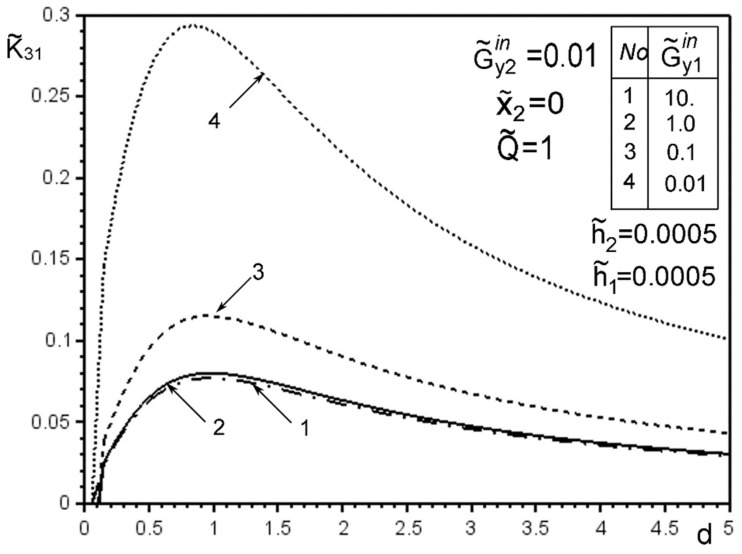
The effect of changing the distance d of the point of application of concentrated forces from the inclusion and the level of dissimodularity to the GSIF ${\tilde{K}}_{31}$ in the absence of surface tension and layer 1 softer from the matrix (4—result for the case of the same layer materials, verified by comparison with [50,53]).

**Figure 11:**
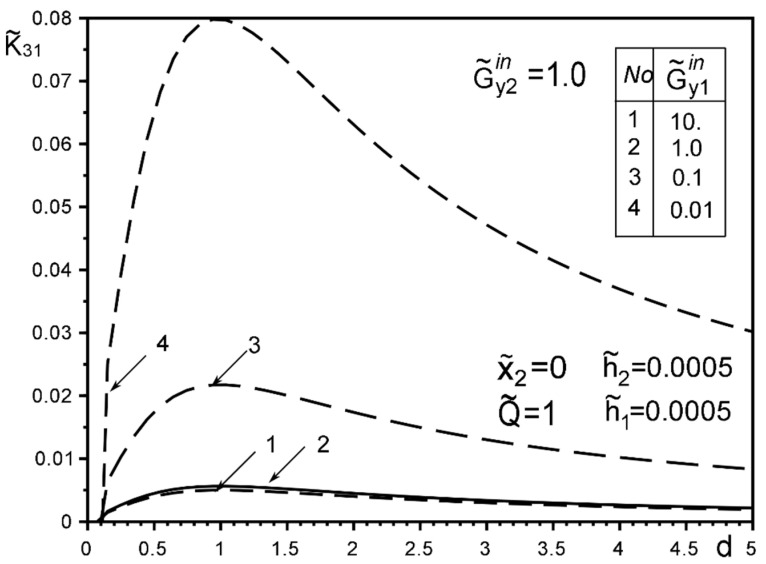
The effect of changing the distance d of the point of application of concentrated force from the inclusion and the level of dissimodularity to the GSIF ${\tilde{K}}_{31}$ in the absence of surface tension and equivalent to the matrix material layer 1 (2—result for the case of the same layer materials, verified by comparison with [50,53]).

**Figure 12:**
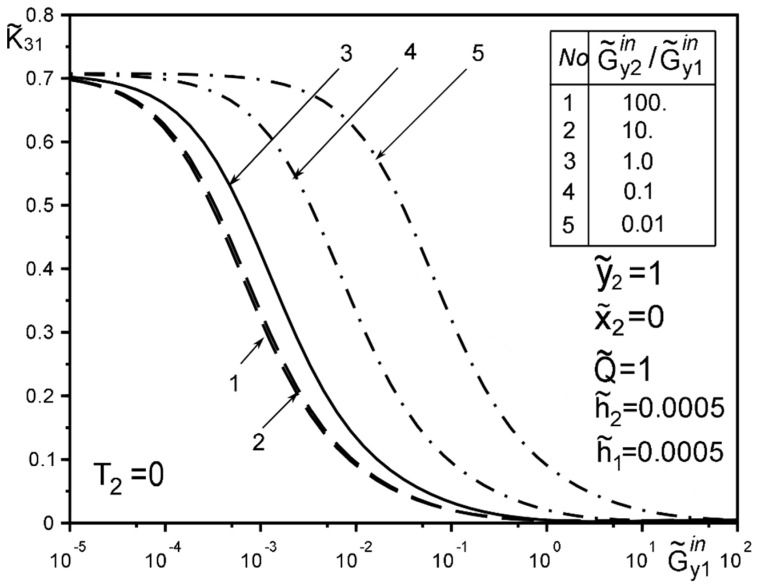
Influence of the level of dissimodularity of the inclusion layer materials on the GSIF ${\tilde{K}}_{31}$ in the absence of surface tension (3—result for the case of the same layer materials, verified by comparison with [50,53]).

**Figure 13:**
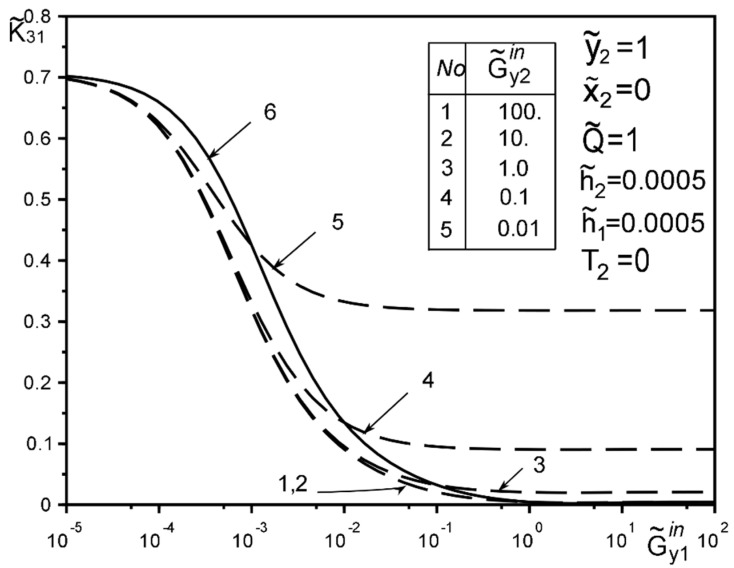
The effect of changing the level of dissimodularity of the inclusion layer materials on the GSIF ${\tilde{K}}_{31}$ in the absence of surface tension under a concentrated force loading.

**Figure 14:**
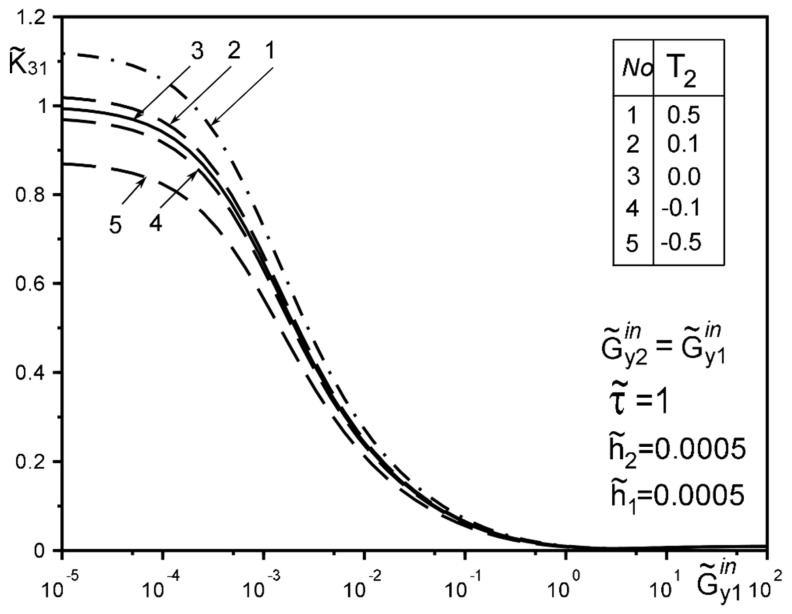
Influence of interlayer surface tension on the GSIF ${\tilde{K}}_{31}$ for the same inclusion layer materials when loaded by a uniformly distributed stress at infinity (3—result for the case of the same layer materials, verified by comparison with [50,53]).

**Figure 15:**
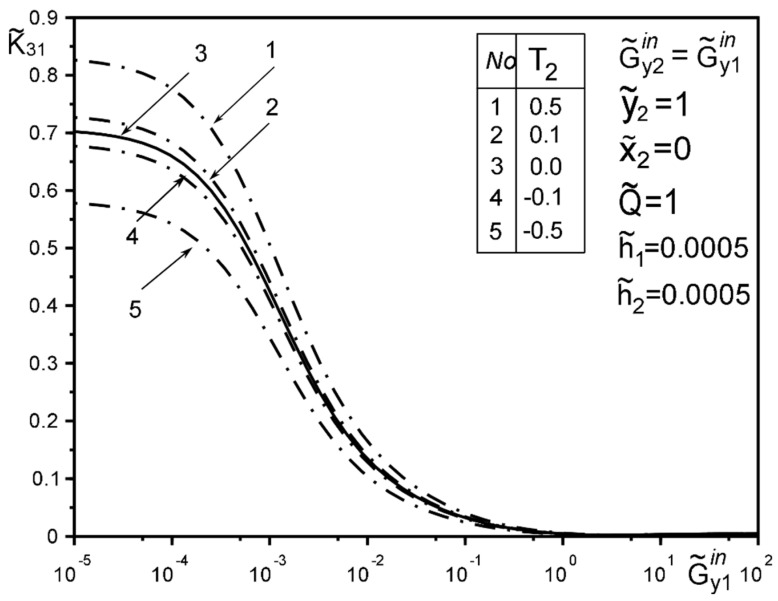
Influence of the interlayer surface tension for the same materials of the inclusion layers when loading with a concentrated force at the point $\varsigma_{2*}$.

**Figure 16:**
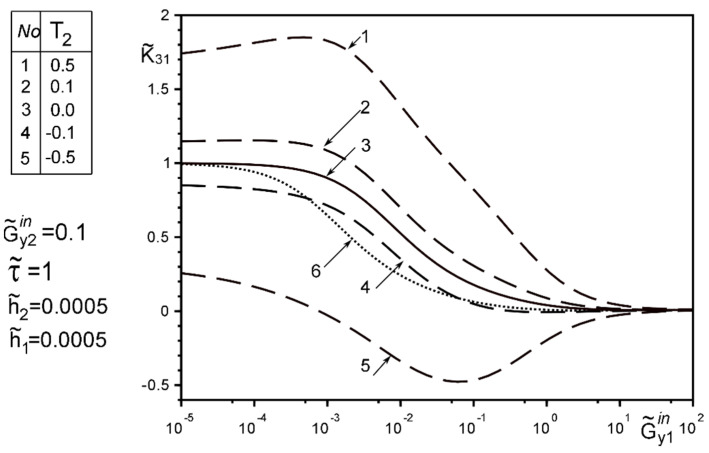
The effect of the interlayer surface tension on the GSIF ${\tilde{K}}_{31}$ when layer 2 is softer than the matrix, and the layer 1 is of the arbitrary material under the loading by stress uniformly distributed at infinity; (6—result for the case of the same layer materials verified by comparison with [50,53])).

The use of dimensionless values will make it possible to interpret the obtained quantitative results and qualitative conclusions on any variant of specific materials of inclusion layers or matrix by simple recalculation due to the universality of the mathematical model of a thin deformable inclusion and the method of problem-solving. The investigation of the influence of the inclusion layers different modularity, external force factors at non-ideal contact with the surface tension of the structural components on the unmeasured stress-strain field parameters on the inclusion surfaces, and the dimensionless stress intensity factor ${\tilde{K}}_{31}$ are illustrated in Figure 4, Figure 5, Figure 6, Figure 7, Figure 8, Figure 9, Figure 10, Figure 11, Figure 12, Figure 13, Figure 14, Figure 15 and Figure 16. Figure 4, Figure 5, Figure 6 and Figure 7 shows the results of a study of the stress distributions on the contact surfaces and displacement jumps on the inclusion as a function of the degree of dissimilarity of the inclusion layers under different external loads (Figure 4 and Figure 5 illustrate the effect of a far-field uniform shear loading, and Figure 6 and Figure 7 illustrate the effect of a concentrated force on similar structures) and in the absence of surface forces.

When one of the layers is significantly softer than the matrix, the effect of “unloading” (stress level reduction) of the surfaces is observed irrespective of the stiffness of the second layer. And this effect is more local than the loading by the concentrated forces located in the points $\varsigma_{k*}=x_{k*}+iy_{k*}$; $x_{k*}=0,y_{2*}=-y_{1*}=d$ of order d/a≈O(1). Figure 4 and Figure 5 reflect the known fact that the stress variation on most of the inclusion surfaces is small and changes abruptly as they approach the tips. In contrast, the applied near the inclusion concentrated force (Figure 6 and Figure 7) essentially perturbs the character of stress distribution along the inclusion axis, its maximum value for such a loading is reached on the geometric symmetry axis of the problem. With the removal of the point of force application (increase in d) the character of the stresses changes approaches the characteristic of a far-field uniform shear loading (Figure 4 and Figure 5). Figure 7d illustrates the proportionality of the displacement jumps of each layer to their stiffness.

Figure 8, Figure 9, Figure 10, Figure 11, Figure 12 and Figure 13 show the results of the study of the effect of the level of dissimodularity on the GSIF ${\tilde{K}}_{31}$ under different external loading and in the absence of surface forces. It is noteworthy that the increase in the level of dissimodularity of the materials of the inclusion layers significantly affects the GSIF when the stiffness of one of the layers is greater than that of the matrix (Figure 8, Figure 9, Figure 12 and Figure 13) regardless of the type of loading.

Figure 10 and Figure 11 confirms the known effect of the GSIF ${\tilde{K}}_{31}$ maximum under the loading by a concentrated forces placed at a distance approximately d≈a from the inclusion axis, irrespective of the stiffness of the materials of the layers. However, it is more pronounced in the stiffness range of the materials of the layers softer than the matrix material. Moreover, if the material of one of the layers is equivalent to that of the matrix (Figure 11), we obtain the known results for a homogeneous elastic inclusion at the interface of the matrix materials [50,51,52,53].

Figure 14, Figure 15 and Figure 16 show the results of the study of the influence of the level of dissimodularity of the inclusion layers and the presence of surface forces under different external loads on the GSIF ${\tilde{K}}_{31}$. It was found that the presence of surface forces leads to an increase in the SIF if they are directed toward the external load, and a decrease if they are directed in the opposite direction from the external load (Figure 14 and Figure 15). The dissimodularity of the materials of the layers significantly distorts this effect, which is especially noticeable when one of the layers is significantly softer than the matrix material. It is revealed that there are certain combinations of external load parameters, surface forces, and material properties of the layers, at which there are local SIF extremes. This effect can be useful in designing the modes of operation of structures with such a structure.

The solution method and results obtained for the two-layer inclusion have been verified by the coincidence of the numerical results with those known in literature [47,50,51,52,53] for a homogeneous thin elastic inclusion—the curves 1 in Figure 5 and Figure 7; 3 in Figure 8; 4 in Figure 10; 2 in Figure 11; 3 in Figure 12; 3 in Figure 14 and Figure 15; 6 in Figure 16.

## 5. Conclusions

A mathematical model of a thin multilayer inclusion of finite length with orthotropic properties of the layers is constructed, taking into account the effect of surface energy on their interfaces. On its basis, we derive a system of equations for solving the problems of antiplane deformation of a bimaterial with thin multilayer interfacial linearly elastic inclusions under arbitrary force and dislocation loading in the case where the inclusion-matrix contact may be ideal or with surface tension or sliding (smooth or frictional).

It is found that for the corresponding problems with bilayer inclusions:

(1) The growth of the level of dissimodularity of the materials of the inclusion layers significantly affects the SIF *K*_31_ when the stiffness of one of the layers is greater than the stiffness of the matrix, regardless of the type of loading. The effect of localization of the maximum SIF *K*_31_, when loaded by a concentrated force located at a distance approximately *d* ≈ *a* from the inclusion axis, is confirmed irrespective of the stiffness of the materials of the layers. However, it is more pronounced in the stiffness range of the materials of the layers softer than the matrix material. Moreover, if the material of one of the layers is equivalent to that of the matrix, then the known results for a homogeneous elastic inclusion (the second layer) at the material interface are obtained.
(2) The presence of surface forces leads to an increasing SIF if they are co-directed with the external load, and a decrease otherwise. The different modularity of the materials of the layers qualitatively changes this phenomenon, which is especially noticeable when one of the layers is significantly softer than the matrix material.
(3) There are certain combinations of external load parameters, surface forces, and material properties of the layers at which there are local SIF extremes.

All these conclusions may be useful for the design of a layered inclusion and the modes of operation of such structures. The proposed method has proved to be effective for solving a whole class of strain problems for bodies with thin deformed inclusions of finite length and may be used for the calculation of FGM inclusions. The addition of the proposed method by the homogenization method [58] will give an obvious opportunity to solve the problem of thin deformable heterogeneous inclusions in periodically layered composites.

## Data Availability

The data presented in this study are openly available at https://doi.org/10.3390/ma14174928, reference number [51], https://doi.10.2478/ama-2018-0029, reference number [52].

## Nomenclature

FGM	functionally graded material;
SIF	stress intensity factor;
SSIE	system of singular integral equations;
SSS	stress-strain state
x,y,z	Cartesian coordinates;
$f_{r}$	jump functions;
$E_{k},\nu_{k},\;G_{k},G_{x}^{inK},G_{y}^{inK}$	elastic properties of the material;
$S_{k}$	half-planes (sections of the body);
$a,h,h_{K},y_{K}$	dimensions of the inclusion layers;
$w,\sigma_{xz},\sigma_{yz},\sigma_{xx},\sigma_{yy}$	displacement, stresses (components of SSS);
$L^{\prime }=\left[-a;\;a\right]$	line, modeling the presence of thin inclusion;
$Q_{k},b_{k}$	magnitudes of concentrated forces and screw dislocations;
$\sigma_{yz}^{\infty }$, $\sigma_{xzk}^{\infty }$, $\sigma_{yy}^{\infty }$,$\sigma_{xxk}^{\infty }$	uniformly distributed in infinity shear stresses;
**Special denotations**	
${\left[•\right]}_{y,h}=•\left(x,y-h\right)-•\left(x,y+h\right)$, $\;{\langle •\rangle }_{y,h}=•\left(x,y-h\right)+•\left(x,y+h\right)$;	
superscripts “+” and “−”	denotes boundary values of functions on the upper and the lower to width inclusion borders accordingly;
superscript “in”	marks the values corresponding to inclusion;
superscript “°”	marks the values in the corresponding problem without any inclusion;
superscript “~”	marks the terms that become dimensionless;
subscript “k”	denotes the terms corresponding to half-plains.

## References

[1] Mura T. (1987). Micromechanics of Defects in Solids.

[2] Paulo Davim J., Constantinos A. (2013). Nanocomposites: Materials, Manufacturing and Engineering.

[3] Wang Y., Huang Z.M. (2018). Analytical micromechanics models for elastoplastic behavior of long fibrous composites: A critical review and comparative study. Materials.

[4] Zhou K., Hoh H.J., Wang X., Keer L.M., Pang J.H., Song B., Wang Q.J. (2013). A review of recent works on inclusions. Mech. Mater..

[5] Mencik J. (1996). Mechanics of Components with Treated or Coated Solids.

[6] Nemat-Nasser S., Hori M. (1993). Micromechanics: Overall Properties of Heterogeneous Materials.

[7] Williams J.C. (1976). Doctor-Blade Process, in Treatise on Materials Science and Technology.

[8] Chen J. (2005). Determination of thermal stress intensity factors for an interface crack in a graded orthotropic coating–substrate structure. Int. J. Fract..

[9] Chen X., Liu Q. (2001). Thermal stress analysis of multi-layer thin films and coatings by an advanced boundary element method. Comput. Model. Eng. Sci..

[10] Elperin T., Rudin G. (2016). Thermal stresses in a coating–substrate assembly caused by internal heat source. J. Therm. Stresses.

[11] Ding S.H., Li X. (2011). Thermal stress intensity factors for an interface crack in a functionally graded layered structures. Arch. Appl. Mech..

[12] Naik R.A. (1992). Simplified micromechanical equations for thermal residual stress analysis of coated fiber composites. J. Compos. Technol. Res..

[13] Perkowski D.M., Kulchytsky-Zhyhailo R., Kołodziejczyk W. (2018). On axisymmetric heat conduction problem for multilayer graded coated half-space. J. Theor. Appl. Mech..

[14] Shevchuk V.A. (2006). Modeling and computation of heat transfer in a system “body–multilayer coating”. Heat Transf. Res..

[15] Ichikawa K. (2000). Functionally Graded Materials in the 21ST Century, A Workshop on Trends and Forecasts.

[16] Kashtalyan M., Menshykova M. (2008). Three-dimensional analysis of a functionally graded coating/substrate system of finite thickness. Phil. Trans. R. Soc. A Math. Phys. Eng. Sci..

[17] Koizumi M. (1993). The concept of FGM. Ceram. Trans. Funct. Graded Mater..

[18] Miyamoto Y., Kaysser W.A., Rabin B.H., Kawasaki A., Ford R.G. (1999). Functionally graded materials: Design, processing and applications. Mater. Technol. Ser..

[19] Zhang N., Khan T., Guo H., Shi S., Zhong W., Zhang W. (2019). Functionally graded materials/an overview of stability, buckling, and free vibration analysis. Adv. Mater. Sci. Eng..

[20] Li Y., Feng Z., Hao L., Huang L., Xin C., Wang Y., Bilotti E., Essa K., Zhang H., Li Z. (2020). A Review on Functionally Graded Materials and Structures via Additive Manufacturing: From Multi-Scale Design to Versatile Functional Properties. Adv. Mater. Technol..

[21] Bishop A., Lin C.Y., Navaratnam M., Rawlings R.D., McShane H.B. (1993). A functionally gradient material produced by a powder metallurgical process. J. Mater. Sci. Lett..

[22] Boch P., Chartier T., Huttepain M. (1987). Tape casting of Al_2_O_3_/ZrO_2_ laminated Composites. J. Am. Ceram. Soc..

[23] Kieback B., Neubrand A., Riedel H. (2003). Processing techniques for functionally graded materials. Mater. Sci. Eng. A..

[24] Mistler R.E. (1973). High strength alumina substrates produced by a multiple-layer casting technique. Am. Ceram. Soc. Bull..

[25] Takahashi M., Itoh Y., Kashiwaya H., Yamanouchi M. (1990). Fabrication and evaluation of W/Cu gradient material by sintering and infiltration technique. Proceeding of the First International Symposium on Functionally Gradient Materials FGM.

[26] Uchida Y. (2004). Properties of functionally graded materials, Manufactured by progressive lamination method for applications. Aichi Inst. Technol. Res. Rep..

[27] Jin Z.H. (2002). An asymptotic solution of temperature field in a strip of a functionally graded material. Int. Commun. Heat Mass Transf..

[28] Lee Y.-D., Erdogan F. (1998). Interface cracking of FGM coatings under steady-state heat flow. Eng. Fract. Mech..

[29] Wang X., Sudak L.J. (2008). Three-Dimensional analysis of multi-layered functionally graded anisotropic cylindrical panel under thermomechanical loading. Mech. Mater..

[30] Yevtushenko A.A., Rozniakowska M., Kuciej M. (2007). Transient temperature processes in composite strip and homogeneous foundation. Int. Commun. Heat Mass Transf..

[31] Yildirim B., Dag S., Erdogan F. (2005). Three-Dimensional fracture analysis of FGM coatings under thermomechanical loading. Int. J. Fract..

[32] Erdogan F., Wu B.H. (1996). Crack problem in FGM layers under thermal stresses. J. Therm. Stresses.

[33] Guo L.C., Noda N., Ishihara M. (2007). Thermal stress intensity factors for a normal surface crack in a functionally graded coating structure. J. Therm. Stresses.

[34] Hsueh C.H. (2002). Thermal stresses in elastic multilayer systems. Thin Solid Film..

[35] Zhuo X.R., Beom H.G. (2016). Interface crack between a thin film and an orthotropic substrate under uniform heat flow. Arch. Appl. Mech..

[36] Bao G., Wang L. (1995). Multiple cracking in functionally graded ceramic/metal coatings. Int. J. Solids Struct..

[37] Moya J.S., Sanchez-Herencia A.J., Requena J., Moreno R. (1992). Functionally gradient ceramics by sequential slip casting. Mater. Lett..

[38] El-Borgi S., Erdogan F., Ben Hatira F. (2003). Stress intensity factors for an interface crack between a functionally graded coating and a homogeneous substrate. Int. J. Fract..

[39] Wang B.L., Mai Y.W., Noda N. (2002). Fracture mechanics analysis model for functionally graded materials with arbitrarily distributed properties. Int. J. Fract..

[40] Zhao J., Silberschmidt V.V. (2005). Microstructure-Based damage and fracture modelling of alumina coatings. Comp. Mat. Sci..

[41] Benveniste Y., Baum G. (2007). An interface model of a graded three-dimensional anisotropic curved interphase. Proc. R. Soc. A..

[42] Chen Y.Z. (2011). Study of multiply-layered cylinders made of functionally graded materials using the transfer matrix method. J. Mech. Mater. Struct..

[43] Gurtin M.E., Murdoch A.I. (1975). A continuum theory of elastic material surfaces. Arch. Ration. Mech. Anal..

[44] Hashin Z. (2002). Thin interphase/imperfect interface in elasticity with application to coated fiber composites. J. Mech. Phys. Solids.

[45] Kim C.I., Schiavone P., Ru C.-Q. (2010). The effects of surface elasticity on an elastic solid with Mode-III crack: Complete solution. Trans. ASME J. Appl. Mech..

[46] Kulchytsky-Zhyhailo R., Matysiak S.J., Bajkowski A.S. (2018). Semi-Analytical solution of three-dimensional thermoelastic problem for half-space with gradient coating. J. Therm. Stresses.

[47] Pasternak I.M., Pasternak R.M., Sulym H.T. (2015). 2D boundary element analysis of defective thermoelectroelastic bimaterial with thermally imperfect but mechanically and electrically perfect interface. Eng. Anal. Bound. Elem..

[48] Pasternak I.M., Sulym H.T., Ilchuk N.I. (2021). Interaction of physicomechanical fields in bodies with thin structural inhomogeneities: A survey. J. Math. Sci..

[49] Peng X.-L., Lee X.-F. (2009). Thermoelastic analysis of functionally graded annulus with arbitrary gradient. Appl. Math. Mech..

[50] Piskozub I.Z., Sulym H.T. (2018). Nonlinear deformation of a thin interface inclusion. Mater. Sci..

[51] Piskozub Y., Sulym H. (2021). Effect of frictional slipping on the strength of ribbon-reinforced composite. Materials.

[52] Sulym H., Piskozub Y., Polanski J. (2018). Antiplane deformation of a bimaterial with thin interfacial nonlinear elastic inclusion. Acta Mech. Autom..

[53] Sulym H.T. (2007). Bases of Mathematical Theory of Thermo-Elastic Equilibrium of Solids Containing Thin Inclusions.

[54] Zhong Z., Yu T. (2007). Analytical solution of cantilever functionally graded beam. Compos. Sci. Technol..

[55] Piskozub I.Z., Sulym H.T. (1996). Asymptotics of stresses in the vicinity of a thin elastic interphase inclusion. Mater. Sci..

[56] Popina S.Y., Sulim G.T. (1987). The limiting load for a brittle body with a thin-walled elastic inclusion. Sov. Mater. Sci..

[57] Sulym H.T., Popina S.Y. (1997). Strength of a body with stochastic distribution of thin defects under the conditions of antiplane deformation. Mater. Sci..

[58] Kaczynski A., Matysiak S.J. (2010). Stress singularities in a periodically layered composite with a transverse rigid line inclusion. Arch. Appl. Mech..

